# A global comprehensive review on cultured seafood

**DOI:** 10.1038/s41538-025-00461-4

**Published:** 2025-07-18

**Authors:** Antonio Borriello, Andrea Pierucci

**Affiliations:** Joint Research Centre (JRC), European Commission, Ispra, VA Italy

**Keywords:** Biotechnology, Economics, Social sciences

## Abstract

A comprehensive review of the current state of research on cultured seafood, which identified 40 articles, focuses on six aspects: technology, public health, consumer acceptance, economic viability, environmental impact, and legislation. Findings suggest that this industry is less mature than the cultured meat counterpart, although there is growing interest by private and public stakeholders. More research is needed to explore the complex interplay of these six domains.

## Introduction

Recent statistics from the Food and Agriculture Organization of the United Nations (FAO) reveal that global per capita fish consumption has reached an unprecedented high, averaging ~20.7 kg per person in 2022^1^. At the same time, according to the last UN estimate, there will be 10.4 billion people on this planet by 2100^2^.

This increasing consumption trend is not only attributed to cultural preferences and lifestyle but also to the economic growth of many nations, allowing for increased affordability and accessibility to seafood products. As the middle class expands globally, particularly in emerging economies, the demand for high-quality protein sources, including seafood, continues to rise. Indeed, poverty headcount ratio ($2.15 a day) has decreased steadily in the past 20 years, reducing from 26.9% in 2003 to just 9% in 2022^3^. These economic and demographic trends are directly contributing to intensified pressures on both marine and aquaculture systems to meet this rising global seafood demands.

Naylor et al.^4^ estimate that when accounting for income growth, the per capita consumption of seafood will double or more by 2050 in some countries (e.g., India, Brazil, Mexico). If an unlikely and radical change in global food consumption behavior does not occur, to sustain current dietary patterns it will be required to increase the global aquatic animal food supply by 22% by 2032, reaching almost 21.3 kg per capita per year^1,5^. This projected increase brings to light the significant challenges faced by the fisheries and aquaculture sectors.

Overfishing, habitat destruction, environmental degradation and health concerns have placed these vital systems under considerable strain, compromising their ability to meet the soaring demand for marine products^6–10^. The precarious state of marine ecosystems is actual, with over 37% of the assessed stocks being fished outside the biologically sustainable levels ^1^. For many others stocks around the world we do not know their actual status ^11^. Both marine and freshwater species like fish, shellfish, and molluscs are at high risk, with over 90% of marine food production globally facing significant threats from various environmental changes, including climate change, pollution, and habitat destruction^7,9^.

Pressures on marine ecosystems continue to increase and intensify, and aquaculture still represents an important component of global food security. However, its expansion brings both opportunities and challenges, necessitating a careful assessment of its environmental and socio-economic implications.

Aquaculture production, has been rapidly increasing since the mid 1980s and surpassed the capture fisheries as a source of food in 2014, and as total production in 2022^12,13^. While some experts caution against excessive reliance on aquaculture, citing declining growth rates and potential risks of fish food shortages^5^, aquaculture production may continue to grow rapidly as the control of the production process allows continued innovation that reduces production costs and improves competitiveness ^13^. The expansion of aquaculture has the potential to help feeding the increasing human population^12,13^, to generate income and employment^14^, to offer valuable ecosystem services, for instance through molluscs and seaweed farming^15–17^, and to reduce pressure on wild fish stocks through advancements in feed efficiency and nutritional improvements^16^. However there are concerns that this rapid expansion of aquaculture can affect its sustainability, in particular its environmental and social dimensions^5,8,18–23^. Feed often determines the growth and quality of the aquaculture production, but also generates the greatest environmental impacts^24^, which are largely dependent on the species cultured and the governance system^17,25,26^. Furthermore, persistent challenges remain, including uneaten feed and feces waste, disease management, escapees, the broader ecological consequences of large-scale production, and the effects of climate change on aquaculture systems^16^.

Amidst the challenges of overfishing and habitat destruction, an innovative approach known as cultured (or cell-based) seafood is emerging as a possible alternative in sustainable seafood production, though doubts remain about whether this technology can truly deliver on its promises or if it risks becoming more hype than solution^27–29^. While this approach offers a groundbreaking alternative to traditional methods, realizing its potential is not without challenges. By cultivating seafood products directly from cells, this innovative method may bypass many of the environmental issues associated with traditional fisheries and aquaculture. Inspired by the successful advancements and substantial investments in cultured meat, cultured seafood could play a positive role in the future of marine and freshwater food production and sustainable management.

At its core, cultured seafood production involves cultivating cells in a controlled environment to develop seafood products without the need for traditional fishing or aquaculture. The process begins with sourcing appropriate cell lines (i.e., cultures of cells that can be propagated repeatedly), which are crucial for determining the species and type of tissue to be produced. These cells are cultured in bioreactors, a closed system that supports cell proliferation through the use of growth media, typically fetal bovine serum or other nonanimal alternatives. The cells grow on a scaffold, which is often edible, to gain three-dimensionality and mimick the structure of real seafood tissues.

While this approach sounds straightforward in theory, its practical application faces numerous challenges. Some of these are mainly technical such as developing stable seafood cell lines, affordable serum-free media, or efficient scaffolding for large-scale production. Others are commercially complex in terms of high costs of production, difficulties in scaling up from lab to industrial levels, and consumers’ skepticism particularly for safety and nutrition. Finally, regulatory approval processes for cultivated (sea)food still represent a big challenge worldwide.

Despite all these obstacles, globally, the cell-based food industry is at a pivotal stage, with numerous startups and established companies racing to bring their products to market and there is a robust global commitment to advancing this innovative field.

Pioneering companies such as BlueNalu and Finless Foods have embraced the promise of cultured seafood, committing to groundbreaking research and development. Simultaneously, governmental bodies are recognizing the strategic importance of supporting research in this innovative field. Initiatives such as the European Commission’s Horizon 2020 program and the United States Department of Agriculture’s competitive grant programs underscore a global commitment to advancing sustainable food production through cellular agriculture. However, the regulation of the industry is not straightforward and only a few countries around the world have moved the first steps in this direction. Investments, regulatory frameworks, and consumer acceptance will have to converge to define the trajectory of this burgeoning industry.

This comprehensive review explores the current state of research in the cultured seafood realm and provides insights into various aspects, including public health, economic factors, and legislative considerations.

### History and status quo

In his essay—Fifty years hence—published in 1931, Winston Churchill summarized the burden of farming and the hope for a more sustainable future by stating that -we shall escape the absurdity of growing a whole chicken in order to eat the breast or wing, by growing these parts separately under a suitable medium-. Fast forward roughly 70 years and Catts and Zurr^30^ prepared two coin sized frog steaks for an art exhibition in France in 2003 as part of a presentation on manipulated living systems in an artistic context. The first product developed for commercial purposes was a cultured beef burger unveiled by Prof. Mark Post at a press conference in 2013. The regulatory landscape for these innovative products has evolved rapidly, reflecting their growing feasibility and public interest. In December 2020, Eat Just became the first company worldwide to receive regulatory approval to sell its cultured chicken in Singapore. Other countries followed suit. In 2023, GOOD Meat and UPSIDE Foods received approval in US to produce and sell cultured chicken; at the beginning of 2024, Israel approved cultured beef produced by the startup Aleph Farms, becoming the first country in the world to regulate a not-chicken cultured product; in March 2024, the Australian company Vow received the approval by the Singaporean authorities to sell cultured quail. Finally, although not for human consumption, UK approved the use of cultured chicken in pet food in July 2024.

**Fig. 1:**
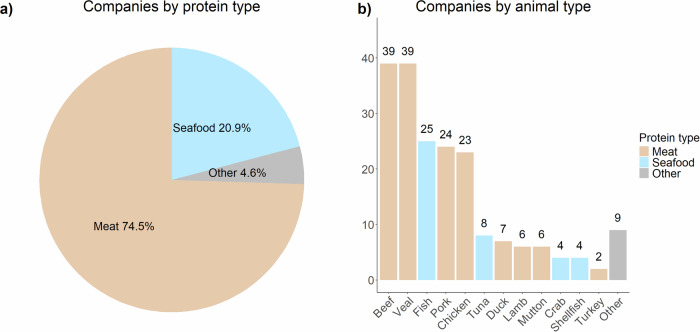
Companies by protein and animal type. Own elaboration based on the GFI database. Note: **a** displays the proportion of companies working on cultured food by protein type. **b** displays the number of companies working on cultured food by animal type.

According to the Good Food Institute, as of April 2024, worldwide there are 112 alternative protein manufacturers that focus on different animal proteins (i.e., meat, seafood, eggs, diary) along different stages of the production line (i.e., cell line development, scaffolding, end product formulation and manufacture, etc.). The disproportion towards cultured meat in the industry is evident, with meat from beef and veal being the most appealing to be lab-manufactured (Fig. 1).

However, in the past few years, cultured seafood has gained interest, and 25 companies worldwide have opened up their labs to research/manufacture this protein type. Of these, 15 focus uniquely on fish products, one on general seafood (e.g., fish, shellfish), and the remaining nine have opened streams of research on seafood alongside those more mature on meat. The first companies working on cultured seafood started up in 2017 (e.g., Finless Food, BlueNalu, Wildtype), but the majority (10 out of 16) started activities from 2020 onwards, marking a 167% increase on a three-years basis. This surge of companies starting up in this domain signals the crescent appeal of the sector. Geographically, 24% (6 out of 25) of the companies have headquarters in the United States and 16% (4 out of 25) in Singapore, reinforcing the link between regulations and marketability.

One of the major disadvantages that these companies face compared to those working on terrestrial/avian counterparts is the limited availability of established and public cell lines. Research on fish cell lines has mainly focused on zebrafish and medaka, with multiple cell lines available for each species. While these fish are not consumed as food, the findings from studying their cell lines could be applicable to other commercially important fish species^31^. The pace of commercial development is mirrored in academic research, where the focus on cultured seafood is beginning to catch up with its terrestrial counterparts, although currently only a few dozen papers focus explicitly on cultured seafood. As a comparison, a review by Kantono et al. in 2022 found 136 articles on cultured meat^32^. The first record of peer-reviewed research on cultured seafood dates back 2002, when Benjaminson et al.^33^ developed a muscle protein production system for a goldfish to help NASA astronauts’ diets during missions.

This paper reviews the existing literature on cultured seafood. The next sections present the existing peer-reviewed research and attempt to classify its contents.

## Methods

**Fig. 2:**
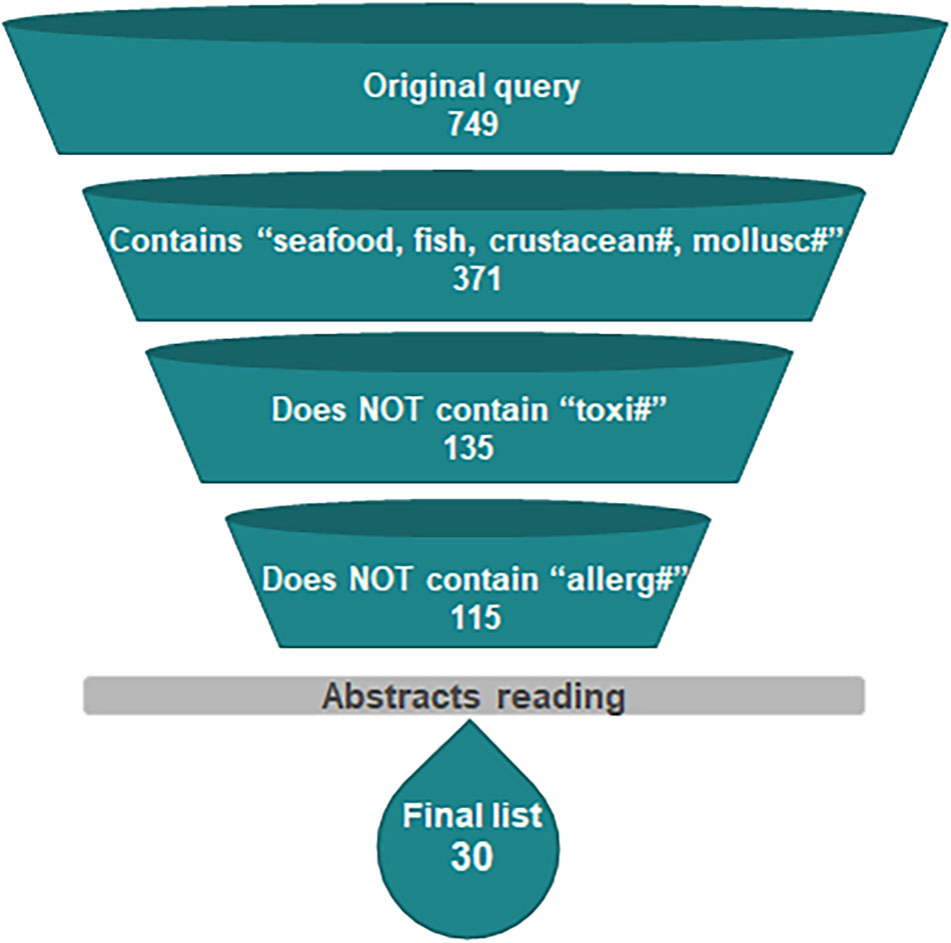
Screening process of the literature search. Note: Initial screening process.

We reviewed the literature on cultured seafood focusing on publications on peer-reviewed journals. Papers that contributed substantially to any aspect of cultured seafood domain were sought on the database Scopus, whilst those that only mentioned this avenue of research in a broader context were not included. The query searched for the following terms: “cell-based seafood’, “cell-based fish”, “synthetic seafood”, “synthetic fish”, “cell-cultured seafood”, “cell-cultured fish”, “cell-cultivated seafood”, “cell-cultivated fish”, “cellular aquaculture”, “cellular mariculture”, “lab-grown seafood”, lab-grown fish”. The search was limited to publications in English and up to 2023 included. The screening process is displayed in Fig. 2.

From the initial search, 749 papers were found. The screening procedure eliminated papers that did not explicitly mentioned the words “seafood”, “fish”, “crustacean”, “mollusc” or any of their derivatives (e.g., crustaceans, crustacea) in the abstract or keywords. A careful analysis of the contents of the keywords of the remaining papers revealed that many of them were related to toxicology and specifically to the ciguatoxin, a toxin involved in a human food poisoning known as ciguatera. These papers were not related to cultured seafood and were therefore excluded. Similarly, papers containing the word “allergy” or any derivatives (e.g., allergies) were also excluded. This screening procedure delivered a set of 115 papers, which were then screened by reading the abstract and, when necessary, other sections of the paper. The final list of papers that qualified for this review focusing on cultured seafood was made up of 30 peer-reviewed articles.

During the review process, the selection of relevant papers was expanded through the inclusion of a second database, PubMed. Additionally, two supplementary search terms, “cultivated seafood” and “cultured seafood,” were incorporated to enhance the comprehensiveness of the review. A targeted search for review articles on the topic was also conducted by screening publications identified using broader terms, including “cell-based food,” “cultured food,” “cultivated food,” “synthetic food,” and “lab-grown food.” As a result of this refined approach, an additional 10 articles were identified, bringing the total number of items that qualified for this review to 40.

It is important to notice that there is a rich literature on the establishment of fish cell-lines for various purposes, including but not limited to aquaculture, pharmacology, disease research and vaccine development. Many of these studies are relevant also for the development of the cultured industry, being cell-lines the essential and basic element to recreate food in a laboratory. However, the majority were not conceived having in mind cultured food as an avenue of research and therefore are not fit for the review.

## Results

**Fig. 3:**
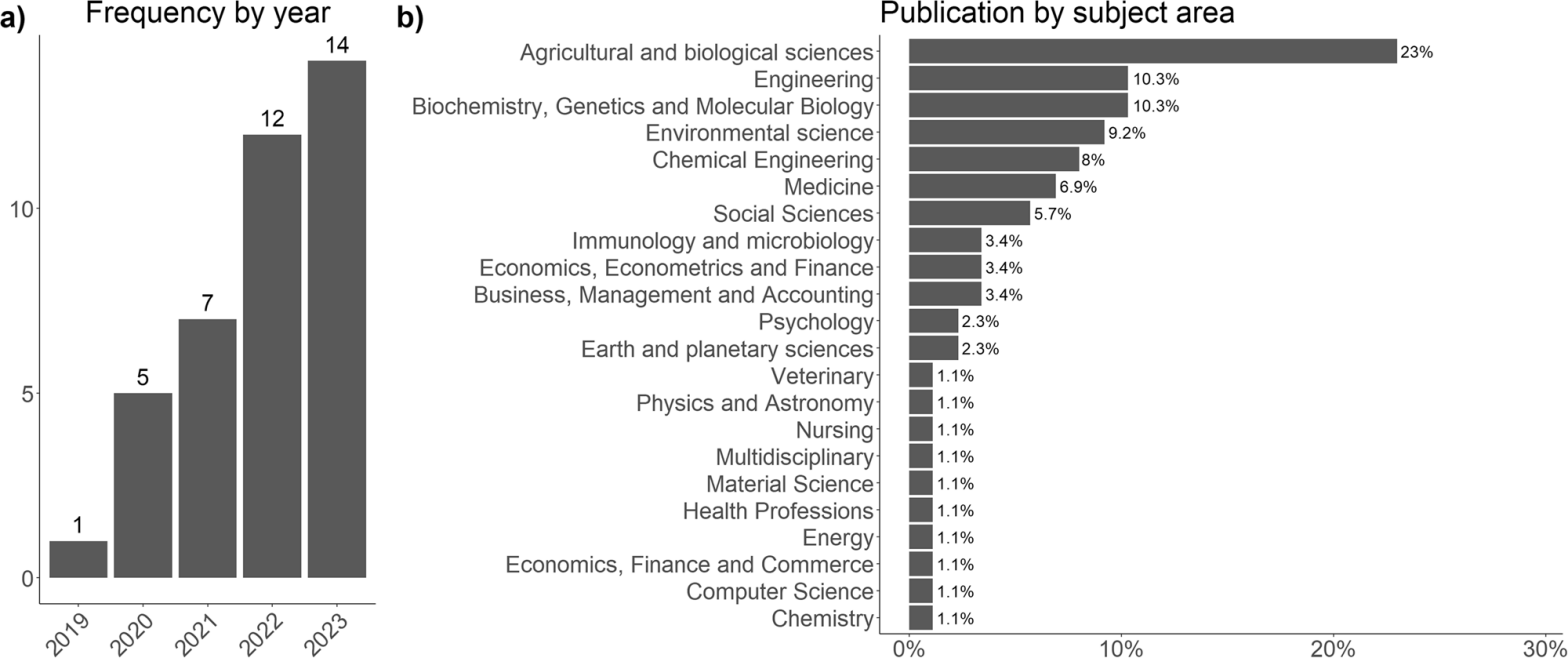
Number of publications in the past five years and by subject areas. **a** Displays the frequency of publications in the past 5 years. **b** Shows the percentage of publications in each subject area.

Although the first publication on cultured seafood dates back to 2002 ^33^, it is not until the past five years that this field of research has gained great interest, pushed mostly by the cultured meat counterpart, which seems to be a few years ahead. This growing popularity is testified by the increasing number of publications, as depicted in Fig. 3.

Most of the papers identified are authors’ perspectives (7) or reviews (20), and as such, their findings and discussions are not specific to any particular country or region, but rather have universal applicability and relevance. For those studies based on a respondents’ sample (in some cases also across multiple countries) or on laboratory analyses, the geographical representation is highly skewed towards US with seven studies. Japan, India and Singapore follow with two, whilst a group of countries is represented with one publication (e.g., Israel, Italy, The Netherlands, UK, New Zealand, Australia, Norway, Brazil, and China).

Figure 3b displays the distribution of the subject areas of the corresponding journals as categorized by Scimago. Note that each journal can list several areas, and some may not be indicative of the study. For example, Ford et al. published their recent study on eating habits on the journal Appetite, which lists both Nursing and Psychology^34^. Most of the papers relate to biological (including genetics and bio-engineering) and environmental sciences, highlighting the relevance and the impact of cultured seafood across both domains.

From a textual analysis of the keywords, it emerges that the most frequent words are “meat”, “animals” and “seafood”, accounting altogether for roughly 7% of the keywords (504 in total across 40 publications, see Table 1). This is of course not surprising considering that these words can be employed in any domain related to the searched query. When looking into details at more specific words, some are peculiar of biology and refer to elements, such as adipocyte, muscle and stem cells, or technical procedures, such as cell differentiations. Interestingly enough, food safety is mentioned several times (5) stressing the relevance of the public and governmental concern for the novel food. Other buzz words not listed in Table 1 but cited several times are “sustainability/sustainable development” (4), “climate change” and “risk assessment” (3).

**Table 1:** Top 15 keywords based on frequency

Keyword	Frequency
Meat	13
Animals	12
Seafood	11
Adipocyte	9
Muscle	9
Agriculture	8
Cell differentiation	8
Mammals	8
Human	7
Fish	6
Adipogenesis	5
Food safety	5
Muscle cell	5
Stem cells	5
Tissue engineering	5
Total	111 out of 504

The remaining sections of this paper address recurrent aspects related to cultured seafood. From the set of papers found on the topic, we have identified the following domains which will be discussed more in details: technological advancements and challenges; public health aspects; consumer acceptance; environmental aspects; economic aspects; and regulatory and legislative aspects.

Table 2 lists the set of papers included in this review and their respective contribution to the six domains identified. Note that some papers touch on multiple domains. The categorization only takes into account the major contributions to specific domains.

**Table 2:** Reviewed papers and contribution to six domains

Authors	Year	Technological advancements and challenges	Public health aspects	Consumer acceptance	Environmental aspects	Economic aspects	Regulatory and legislative Aspects
Benjaminson et al.^33^	2002	x					
Rubio et al.^35^	2019	x					
Miller ^64^	2020	x	x	x			
Potter et al.^42^	2020	x	x			x	
Hallman and Hallman ^74^	2020			x			x
Santo et al.^62^	2020		x		x	x	x
Choudhury et al.^50^	2020	x				x	x
Halpern et al.^78^	2021				x		
Ong et al.^83^	2021						x
Reis et al.^79^	2021				x	x	
Waltz ^93^	2021	x				x	
Lonkila and Kaljonen ^49^	2021			x			x
Hallman and Hallman ^75^	2021						x
Lee et al.^41^	2021	x					
Carneiro et al.^71^	2022			x			
Chong et al.^44^	2022	x					
De Amstalden ^91^	2022						x
Goswami et al.^36^	2022	x	x				
Knežić et al.^40^	2022	x					
Lindfors and Jakobsen ^81^	2022					x	
Marwaha et al.^66^	2022		x		x	x	
Sugii et al.^43^	2022	x					
Tsuruwaka and Shimada^48^	2022	x					
Bomkamp et al.^56^	2022	x					
Fernandes et al.^94^	2022	x		x	x		
Ye et al.^53^	2022	x	x	x		x	x
Lamy and Bomkamp^37^	2023	x				x	
Bomkamp et al.^95^	2023	x					
Braun and Knight^69^	2023			x			
Ford et al.^34^	2023			x			
Goswami et al.^36^	2023	x					
Guo et al.^55^	2023	x				x	
Ong et al.^90^	2023						x
Saad et al.^46^	2023	x					
Sugii et al.^63^	2023		x				
Telesetsky ^80^	2023				x		
Xu et al.^47^	2023	x					
Giacalone and Jaeger^77^	2023			x			
Azhar et al.^38^	2023	x					
Santos et al.^52^	2023	x	x				x

### Technological advancements and challenges

The production of cultured seafood is a promising yet complex technology that requires significant advancements across multiple domains. This section aims to outline the current state of the technology and the associated challenges rather than delving into the technicalities of the processes involved.

The majority of research on cellular biology has focused on terrestrial and avian species, leaving a significant gap in the knowledge of aquatic and marine organisms, particularly crustaceans and mollusks^35,36^. As a result, the cultured seafood industry faces significant challenges, including the lack of established protocols. Essential resources, such as sophisticated genome annotations and species-specific reagents (e.g., antibodies) for seafood-relevant species, are either limited or not commercially available^37^. The scarcity of scientific literature in this field pushes private companies to make significant up-front investments in research and development ^37^.

Cell line development serves as the foundation for cell-based food production. Establishing stable cell lines is crucial, as primary cells typically stop proliferating after 30–40 passages, yet this process remains a significant challenge^38^. Some embryonic cell lines of aquatic animals have already been developed and are available at repositories, such as the ICAR-National Bureau of Fish Genetic Resources in India, while private companies, such as Kerafast, have also established cell lines for various species, including fish^38^. Notably, many fish species exhibit high expression of the enzyme telomerase across multiple tissue types, which may enhance cell proliferation and facilitate the development of immortalized cell lines for both research and commercial applications^39^.

For the manufacture of cultured seafood, pluripotent stem cells (i.e., embryonic, adult and induced pluripotent) and multipotent (i.e., mesenchymal, adipose tissue-derived, fibro-adipogenic progenitors, resident muscle) stem cells are relevant. All stem cells have the ability to self-renew, propagate and to differentiate into many cell types (e.g., muscle and/or fat cells, chondrocytes or fibroblasts). Unfortunately, most of the studies on the efficient isolation and in vitro maintenance of these cell types are not oriented towards the food production as they miss 3D culture aiming at engineering a tissue^40^. For the production of cultured food, pluripotent stem cells offer great versatility but are highly sensitive to their growth environment, making them difficult to handle. In contrast, multipotent stem cells-obtainable through biopsies from any animal species-require a scaffold substrate for anchorage and have a more limited proliferative capacity^38,41^.

When cell lines are derived from fish muscle, they are often not well-characterized as muscle cells or fail to demonstrate long-term viability in culture, limiting their applicability to cultured seafood production. There is a clear imbalance in cell-based studies, as the fish species most frequently studied, such as zebrafish, are not widely consumed, reducing their direct relevance to large-scale seafood production. Some authors^42^ suggest starting simpler by developing cultured seafood via a lean fish, such as the zebrafish, due to the technical challenges of co-culturing fat and muscle cells, which is critical to the creation of a product that accurately replicates the texture and flavor of traditional seafood. Zebrafish represents a strategic starting point for research and development, offering the most expedient route to creating full-tissue, cultured fish meat^42^.

Most of the research focuses on muscle cultivation, which is the primary building block of cellular foods, although fat is an essential component for flavor, texture, and nutrition in food products^43^. Some examples include a muscle-derived cell line initiated from the tail of a juvenile snapper^44^, a muscle tissue of Labeo Rohita, a widely cultivated tropical freshwater carp^45^, and Atlantic mackerel muscle cell^46^. Instead, studies on adipose tissue biology have been sporadic at best^47^, and it is hard to find fish cell lines available for investigating adipogenesis^43^. Recently, it has been shown that fish fin cells have the potential to differentiate into various cell types, such as muscle, fat, fibers, and nerves, through treatments simpler than those required for mammalian cells^48^. These differentiated cells could be manipulated and arranged in different proportions to create structured, cultured seafood products.

Evidence suggest that fish cells may offer several advantages over mammalian and avian cells, including better resilience in oxygen-limited environments, tolerance to a broader range of pH levels, and the ability to grow at lower temperatures^35^. For example, fish cells have demonstrated the ability to maintain karyotypic stability (i.e., the ability to maintain chromosome number from doubling to doubling) and replicate effectively in atmospheric air without the need for added carbon dioxide in bioreactors^42^.

Once cell lines are established, they are transferred into bioreactors where they proliferate with the help of a growth medium. Growth media present one of the biggest technical and economic challenges in this process. The most commonly used medium today is Fetal Bovine Serum (FBS), a nutrient- and protein-rich liquid derived from fetuses taken from pregnant cows. However, FBS is not a viable option for many companies due to economic and ethical concerns^49^, and many have expressed intentions to adopt non-animal components. Some companies, such as Blue Nalu, have already achieved this goal, although the exact composition of their alternative media is generally kept confidential^50,51^. Benjaminson et al. demonstrated that a crude extract from maitake mushrooms produced similar results to FBS when used to grow fish primary cell explants^33^. Further optimization of serum-free media has been achieved through microbial fermentation to produce recombinant growth proteins^50^. Santos et al. provide a detailed list of animal-free media currently in development, which include bacteria-based supplements, okara, and yeast extract^52^.

Current bioreactors used for cell expansion and differentiation in meat cultivation have limited volumes, which restrict large-scale production. While some companies have recently made progress in accelerating the production of cultured products (e.g., Wildtype thanks to the commercial agreement with Solaris Biotech), the initial cultured meat samples were produced using multiple small bioreactors, a process that is both labor-intensive and costly. Scaling up bioreactor volume remains a significant challenge due to the need to manage physical and biochemical parameters (e.g., shear stress, pressure, temperature) to ensure proper cell proliferation^53^.

Another relevant consideration for the technical manufacture of cultured seafood is the three-dimensionality of the final product. For cells to grow in an in vitro environment, it is crucial to engineer a scaffold that mimics the natural microenvironment, allowing cells to adhere properly. This scaffold will support the entire cellular process, from cell seeding to the final product. It should be biodegradable, edible, safe for consumption, palatable, and capable of efficiently exchanging gases, nutrients, and waste^38,54^. Notably, when the thickness of conventionally cultured muscle cells exceeds 200 μm, the inner cells begin to die due to insufficient oxygenation and nutrient supply^54^. Current scaffolds that avoid mammalian-derived biomaterials often use substances like salmon gelatin, alginate, cellulose, starch, chitin, chitosan, hyaluronic acid, fibrin, collagen, keratin, and silk^38^. Salmon gelatin, in particular, is a promising scaffold material due to its physical properties, which allow it to be blended with other biopolymers.

Several scaffold fabrication techniques exist, but each comes with its limitations^41^. For instance, electrospinning, which mimics the extracellular matrix by creating ultrafine fibers from a polymer solution using an electric field, results in small pore sizes and thin cells, complicating cell differentiation and proliferation. Mold casting and injectable systems, where liquid materials (e.g., hydrogels, biopolymers, or collagen) are injected into molds to create the desired shape and structure, offer limited control over scaffold pattern porosity and properties. Although it is still an underdeveloped area, the use of 3D bioprinting seems very promising. This technology can potentially overcome some of the current limitations in scaffold development by allowing for the precise layering of cells and biomaterials to create complex biological structures. Despite its potential, there are significant hurdles to be addressed, including the development of edible biomaterials that can be used in 3D bioprinting and the need for more research focused on food products beyond beef and pork^55^. A successful example of this process is a cultured fish fillet (i.e., yellow croaker) derived with 3D bioprinting from piscine satellite cells and piscine adipose-derived stem cells^47^.

The tissue structure of many seafood species is simpler compared to terrestrial animals, which typically have complex, intricate vasculature and marbling. This relative simplicity may make it easier to create a scaffold that replicates the natural composition of the animal. However, more control over the structural formation is necessary to ensure that cultivated fish meat closely resembles its conventional counterpart, minimizing perceptible differences^37^. Considering the heterogeneous approach taken by the different companies within the industry, it is unlikely that a single approach will be suitable for all types of production; instead, a range of solutions will likely be required to meet the needs of different end products^56^.

### Public health aspects

Conventional seafood represents the most valued food for its content of essential nutrients, particularly omega-3 fatty acids—which are beneficial for heart health^57^, selenium, vitamin D and vitamin B12^58^. Nevertheless, conventional seafood may also hide health-hazardous contaminants^42,59^ and pathogens^60^.

The ability to produce seafood in a controlled, sterile environment through cellular aquaculture could eliminate many of the environmental pollution risks of common seafood, such as heavy metals (mercury, lead, chromium, cadmium, and arsenic), microplastics, microfibers^61^, nanoplastics and many others by creating a product free from environmental contaminants and pathogenic microorganisms^36^. Through precise control over the cellular growth environment, it is even possible to enhance the production of the desirable fatty acids—such as eicosapentaenoic and docosahexaenoic—while minimizing the presence of less beneficial fats^62,63^. This level of control could result in seafood products that not only replicate the sensory experience of traditional fish but also offer enhanced nutritional benefits, potentially aiding in the prevention of diet-related chronic diseases and promoting better public health overall^63^. However, challenges may arise in ensuring nutritional validation. Unlike conventional seafood, where nutrients are regulated by the organism’s intake, digestion, and systemic homeostatic mechanisms, ensuring a balanced and consistent nutrient composition in the animal’s flesh, the nutrient content in cultured seafood depends on the growth medium. While this medium can be designed to maximize cell growth, the absence of the whole-animal homeostatic mechanisms means that the nutrient profile of cultured seafood may differ from that of traditionally harvested fish. As a result, there is a need for rigorous validation of the nutrient, micronutrient, and amino acid content of cell-cultured seafood, as well as its nutritional bioavailability and protein functionality, to ensure it meets or exceeds the nutritional standards of conventional seafood products^64^.

Another potential benefit of cultured seafood relates to its traceability. Indeed, where common seafood eco-label certification schemes fails or face problems^65^, cultured seafood production may offer a new and robust support to the issue of food fraud, where consumers are misled about the origin, species, sustainability or quality of seafood products. This type of fraud not only deceives consumers but can also expose them to unexpected allergens or lower nutritional value products. By ensuring that the production process is transparent and traceable, cultured seafood can reduce the risk of such fraudulent practices, thereby protecting consumer health and confidence^66^.

Although the adoption of seafood cell-based technologies has often been presented with potential public health benefits, some concerns arise due to the novelty and the secrecy of the process in play. For example, genetic stability in cultured cells is not fully controllable, and it remains uncertain whether these cells can maintain stability and avoid mutations when cultured at high density in large-scale bioreactors. Moreover, cultivated cell lines are generally not designed for human consumption. Immortal cell lines, in particular, may express cancer-related genes either through spontaneous changes or genetic modification. As a result, it is crucial to ensure that food products derived from these cells do not pose a tumorigenic risk^52^.

In addition to genetic concerns, the growth of these cells relies on medium and serum, which are vital for their proliferation and differentiation. However, these ingredients lack a history of use in food production and therefore require thorough safety assessments, alongside other materials like microcarriers and scaffolds^53^. Furthermore, because raw cultured tissue is nearly colorless, food colorants are necessary to improve its appearance. The long-term health risks of consuming cultured meat also need further investigation^53^.

**Fig. 4:**
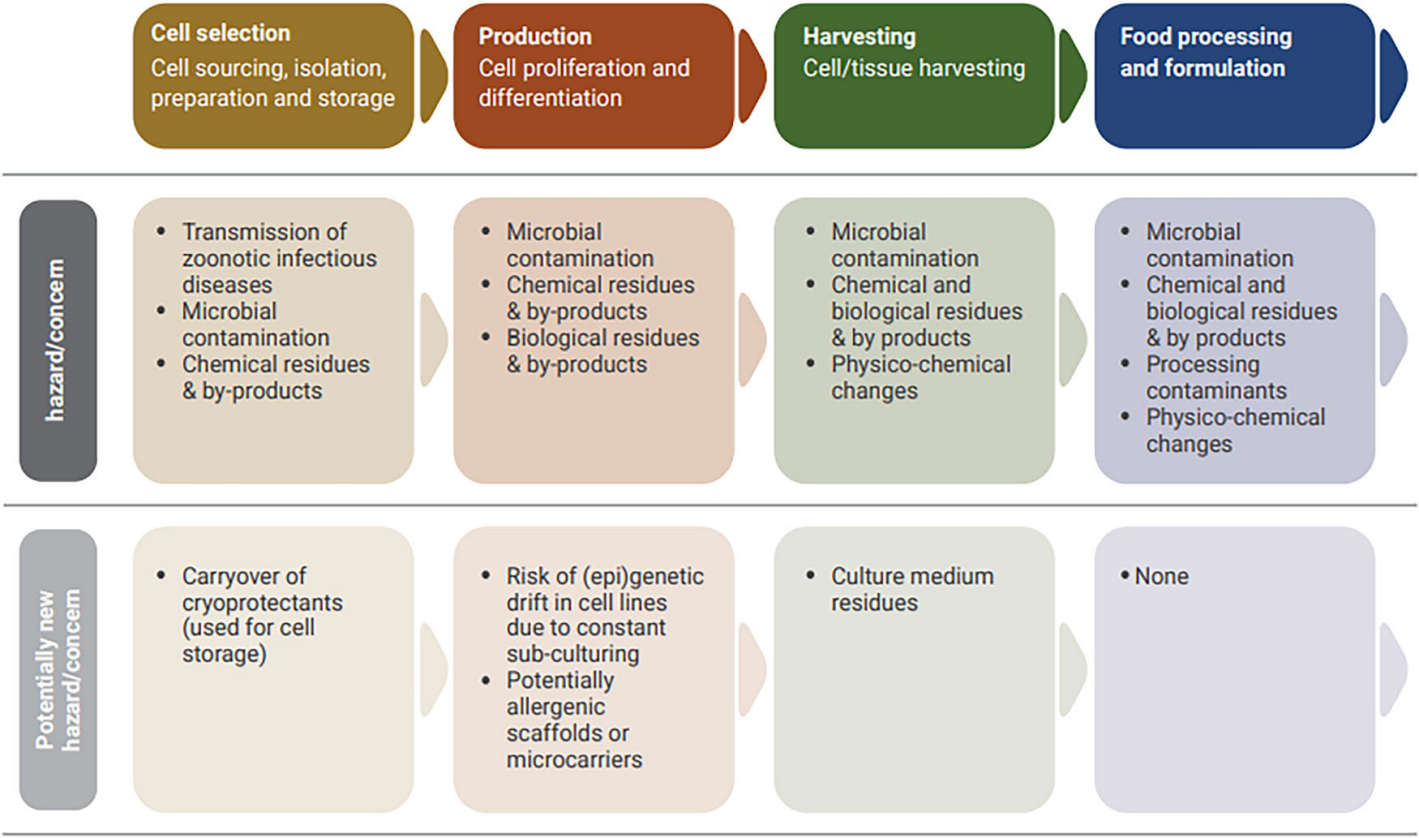
Examples of safety hazards by stage and type. Note: Food safety hazards by cell-based food production phase Source: ^67^.

In a recent report by FAO and WHO^67^, a pool of experts categorized potential hazards and risks of this novel approach throughout four different stages, namely cell-selection, (cell growth and) production, harvesting, and food processing and formulation. In total, the experts found 19, 13, 11, and 9 potential hazards in the four stages, respectively. However, it should be noted that many hazards are already well-known and they exist in conventionally produced food. Figure 4 depicts examples of potential food safety hazards and concerns at different phases of cell-based food production, distinguishing between conventional and new hazards.

Finally, it also remains to be seen whether potential antibiotic use and waste management practices associated with cultured meat production will impact people who work on or live near production facilities, as they do with industrial food animal production^62^.

### Consumer acceptance

Consumer acceptance is a critical factor that may determine the success and future of cultured seafood in the marketplace^68^. While there is substantial research on consumer acceptance of cultured meat, studies specifically focused on cultured seafood are limited. Consumption patterns for conventional meat and seafood are highly variable across markets, and this heterogeneity could be maintained also for the cultured products. However, given the similarities in their production processes, we base this section on the assumption that consumer responses to cultured seafood will align with those observed for cultured meat, as suggested by Ford et al.^34^.

Several elements influence whether consumers are willing to adopt this novel food product, such as sensory attributes (e.g., flavor, texture, feeling of unnaturalness), psychological (e.g., neophobia, anticipated high prices) and cultural factors (e.g., ethics and religion)^34,69,70^. To address these issues, the industry is focusing on developing products that closely mimic the taste, texture, smell, and appearance of traditional seafood^71^.

One of the primary factors affecting consumer acceptance in food choice is the visual appeal, as consumers often associate the color and appearance of meat with freshness and quality. For example, the market value of salmon, red porgy or red sea bream is dependent on the color of the fish, so is on the exoskeleton and muscular ephitelium for shrimps, lobster and other crustacean carapaces and molluskan gonads^72^. The conversion process, in which muscle cells are transformed into an edible product, must be carefully managed to maintain proper pH levels and visual cues of freshness while ensuring the final product closely resembles conventional seafood ^64^. Also, achieving a sensory equivalence between the natural and the lab-made products is crucial, as consumers are more likely to accept and pay a premium for cultured seafood if it delivers a familiar eating experience^34,71^. Other factors could play a determinant role in shaping consumer preferences and could be employed by the industry to increase its appeal, such as nutrition customization and contaminant elimination, for which consumers could be willing to pay a premium^73^.

Another important consideration is the naming and labeling of cultured seafood as the terminology used on packaging can significantly impact consumer perceptions and acceptance^74^. The term “cell-based seafood” has been identified as the most favorable option, as it meets regulatory requirements, avoids consumer confusion, and accurately reflects the production process^75^. Moreover, this label effectively communicates potential allergenicity and the technological nature of the product, which can help build consumer trust. Furthermore, labels highlighting contaminant-free and sustainable qualities enhance consumers’ willingness to pay for cultured seafood, helping the industry position itself as a safe and sustainable option^73^.

Research is still scarce and there is no consensus on which, whether and to what extent attitudes, emotions and perceptions are linked to consumer preferences for this novel food type. Consumer attitudes toward cultured seafood vary across different demographics and regions. For instance, a study conducted among Japanese residents found that while awareness of cultured seafood was relatively low, attitudes-particularly among younger individuals-were generally positive. Approximately 70% of participants expressed interest in trying cultured seafood, and 60% indicated a willingness to purchase it. Interestingly, prior knowledge of the product was strongly linked to a willingness to pay a premium, suggesting that increasing consumer education could be a key strategy for enhancing acceptance^69^.

When comparing different production methods (i.e., fishing, farming, manufacturing in a lab) for the same type of food (tuna, salmon and shrimps), American consumers exhibited a stronger preference for cultured production methods over farm-raised options, primarily due to concerns about contaminants^73^.

However, it is important to note that, compared to other novel food types, cell-based foods, including seafood, often face greater resistance and less interest and trust^76^. Research^77^ shows that consumers exhibit the lowest willingness to consume cell-cultured food products, with significant emotional barriers such as the perception of novelty and concerns over sustainability influencing their decisions. While novelty can drive some consumers to try cultured seafood, the perceived sustainability of these products may also play a crucial role in long-term acceptance^77^.

Ultimately, consumer acceptance will determine whether the industry has a viable future^53^ and therefore it is suggested that as much effort will be put into understanding emotional and attitudinal aspects of consumers as for technical aspects such as flavor, texture and visual characteristics.

### Environmental aspects

Environmental impacts of cellular agriculture are not yet thoroughly documented in the literature, and even less frequent is research specific on cultured seafood products. Proponents of this technology suggest that it has the potential to alleviate some of the sustainability concerns associated with conventional seafood production, such as overfishing and habitat destruction. Indeed, companies like Wildtype, Umami Bioworks, Bluu Seafood and many others published mission statements along these lines on their website. This novel industry offers an alternative to wild-caught and farmed fish, and has the potential to alleviate pressure on wild fish populations, contributing to the broader conservation of ocean habitats. Halpern et al.^78^ outline a sequence of nine critical steps that must be implemented to achieve meaningful marine conservation. They emphasize that any disruption or failure in executing any of these steps could significantly undermine, or even nullify, the anticipated conservation benefits associated with this industry. For the authors^78^, currently cultured seafood has a low chance to lead to meaningful fisheries recovery and ocean conservation benefits.

Cultured seafood may decrease the environmental impact associated with the global seafood supply chain. Traditional fishing often involves transporting fish over long distances, contributing to significant carbon emissions. For example, a substantial portion of tuna caught in regions such as Africa, South America, and the Pacific Islands is shipped to markets in the European Union, a process that involves considerable energy expenditure. By producing seafood closer to consumer markets, cellular aquaculture could lower the carbon footprint associated with seafood transportation^79^.

However, these potential environmental benefits of cultured seafood are not without caveats. The technology’s environmental impact, particularly in terms of energy and resource use, remains a subject of debate. Current literature suggests that cultured seafood may require more land and freshwater than traditional fisheries, although less than conventional aquaculture^66^. Additionally, life cycle assessments have highlighted that the global warming potential of cultured seafood, especially when produced using conventional energy sources, may be higher than that of both wild-caught and farmed fish. This is largely due to the substantial energy demands of bioreactors, which are essential for cellular production^80^. It is to be noted that cultured seafood production requires in theory less energy than meat due to its physical and biological properties (i.e., ability to grow at lower temperatures)^35^. Finally, the potential environmental impact of advancing technology or the integration of the technology with green energy sources remains uncertain.

### Economic aspects

The economic viability of the cultured seafood industry is a significant challenge, marked by exceptionally high production costs that hinder its ability to compete with traditional seafood products. These costs are rapidly decreasing as technology advances.

In 2013, Mosa Meat produced the first burger patty at a cost of $330,000; in 2019, Shiok Meats produced shrimp dumplings at a staggering $5000 per kilogram, while Wildtype’s salmon sushi roll cost ~$200. Currently, GOOD Meat advertises its products from roughly $3 to $18 per serve, depending on the venue. These costs are primarily driven by extensive research and development efforts needed to establish viable cell lines for various species, and by the high expense of the growth media required for cell culture^51,81^, which is the most expensive factor in the production cost. In 2023, in a world’s first, GOOD Meat received approval by Singapore Food Agency for the production of cultured meat without the use of serum, marking a pivotal step towards the potential reduction of this cost category. Other relevant costs relate to the purchase of large bioreactors and processing equipment and to labor, which is highly specialized and composed among others by engineers and microbiologists^51,62^.

Depending on variables such as the cost of growth factors and recombinant proteins, it is estimated that it costs from $150 to $22,423 to produce one kilogram of generic cultured meat^82^. Technological advancements like 3D bioprinting, however, hold promise for reducing these costs by lowering labor expenses through automation, minimizing waste, and improving production efficiency, potentially making cultured seafood more price-competitive with conventional meat^55^. When considering lower prices for growth factors and recombinant proteins, social investments, shorter production run time as well as a larger cell volume, the cost to produce 1 kg of generic cultured meat could be as low as $6^82^.

The financing of the cultured meat industry largely relies on private investments^83^, particularly from venture capital. Very large players of the food industry are shifting their businesses from meat, seafood, dairy and eggs to the macronutrient *protein*. For example, the global leader in aquaculture of pangasius Vinh Hoan (turnover of USD 411 million in 2023)^84^ has been expanding its operations into cultured seafood, making notable investments in startups like Avant Meats—creator of the world’s first cultured fish fillet—and Shiok Meats—the world’s first cultivated crustacean meat company. Similarly, Pulmuone, a leading South Korean food company with a reported sales of nearly USD 2.21 billion in 2023^85^, that specializes in tofu among other products, has invested in BlueNalu - an American company focusing on cultured bluefin tuna—as well as in a South-Korean food-tech startup, Simple Planet. The interest of big players may affect the cell-based industry, which could be monopolized by dominant firms^86^. This could exacerbate the economic and power imbalance in the food system across countries, with low- and middle-income countries (LMICs) suffering the most. However, in regions where traditional aquaculture remains highly profitable (e.g., China, India, Indonesia, Viet Nam etc) ^5^, the immediate interest in cell-based alternatives may be limited due to the distinct knowledge base and innovation paths of the two industries, making resource sharing and collaboration challenging^81^.

While private investments have accelerated growth, they have also created a lack of transparency in technological progress, potentially slowing the industry’s overall development. Indeed, an over-reliance on private funding might drive companies to create and protect intellectual property, limiting collaboration and information-sharing, both critical for addressing technical and economic challenges. The business model in this emerging industry often depends on establishing strong entry barriers through patents, trade secrets, and regulatory constraints, which can aggravate centralization and increase vulnerabilities to disruption^87^.

Although a minority, some public direct and indirect investments were carried out in several countries around the world. The most important of these investments were promoted by The Singapore Food Agency in 2019 and by the Dutch government in 2022, who announced €100M (roughly $108M) and €60M (roughly $65M) for research related to cultured meat, respectively^53^. Other relevant public funding were carried out in UK, US, Israel, Singapore, Japan, China, Israel, Belgium, and New South Wales (Australia)^53,88^.

The economic implications of the cultured seafood industry extend beyond production costs and funding sources. Large-scale production of cultured seafood could lead to significant changes in global supply chains. For instance, the reduced reliance on wild-capture fisheries might promote more localized production, decreasing the need for extensive product transportation^79^. Large fish-processing companies may also enter this innovative market while continuing to produce conventional seafood, potentially benefiting from more stable supply and consistent product quality due to the reduced effects of seasonality on fish stocks^79^.

Finally, the cultured seafood industry could create displacements also along the chain, particularly in LMICs. This is true especially in upstream sectors such as vessel construction and fish processing^66^. Downstream sectors, like packaging and transportation, may face less disruption as these processes are expected to be similar for cell-based products.

### Regulatory and legislative aspects

As of now, no country has authorized the sale of cultured seafood, while only a few have granted approval for cultured meat products. The regulatory landscape for cell-based food is evolving, with significant variability across regions, reflecting the complexities of developing appropriate frameworks for novel food products. Regulatory approvals are crucial for market access, yet standardized best practices or technical guidelines for consistent international regulation are largely absent. Existing frameworks often lag behind the rapid advancements in alternative proteins, including cultivated meats, plant-based products, and fermentation-enabled proteins, leading to a patchwork of regulations that varies significantly by country. Because of the fragmentation of the regulations worldwide, companies have to seek approval in each market they are planning to sell their products, which extends the time to market for products. Different regions may impose varying requirements, from basic product declarations to more stringent guidelines that distinguish cultured from conventional seafood^74^.

Singapore has been a pioneer in this field, being the first country to approve a cultivated meat product in 2020, thanks to the Singapore Food Agency. In 2023, Singapore again led globally by granting regulatory approval for the use of serum-free media in the production of cultivated meat to GOOD Meat. These milestones highlight Singapore’s proactive approach and trust for the success of this industry.

In contrast, regulatory developments in other countries have been slower. China’s Ministry of Agriculture and Rural Affairs included the intention to invest in cell-based food technologies in its 2021 five-year plan, but no specific regulations have been implemented yet. Similarly, Japan lacks a clear legal framework for Novel Foods, creating challenges for establishing and enforcing regulations in the cell-based food industry. Australia and New Zealand operate under a joint food regulatory framework through the Food Standards Australia New Zealand (FSANZ). FSANZ regulates cultivated products under its existing novel foods framework, which includes a pre-market safety assessment to ensure product safety before they reach consumers.

In the European Union (EU), the Novel Foods Regulation sets out the requirements for pre-market authorization of cell-based foods. The European Food Safety Authority conducts risk assessments and provides scientific opinions that inform the European Commission’s final approval decisions. However, in the absence of a general EU-wide approval, individual Member States have taken divergent actions. For instance, Italy banned the production, use, and commercialization of cell-based products in late 2023^89^, and other countries like Romania, France, and Austria are considering similar bans.

The United States employs a dual-agency approach to regulation. The Food and Drug Administration oversees cell collection and all processes up to the “harvest” stage, while the Department of Agriculture handles the processing, packaging, and labeling stages. Despite federal oversight, some U.S. states, such as Florida and Alabama, are pushing for bans on cell-based products, similar to actions taken in parts of the EU.

The current reliance of regulatory agencies on industry-provided data, rather than independent academic research, raises concerns about the comprehensiveness of safety assessments. While broad guidance for safety assessments exists, it often lacks the detail needed to keep pace with fast-evolving technologies in the sector. Industry stakeholders and scientists emphasize the importance of clear understanding and regulation of what goes into the final product, the nutritional profile of cell-based foods, and standardized production methods to facilitate regulatory monitoring and ensure consumer safety^83,90^.

A key regulatory challenge for governments worldwide is the labeling of the manufactured products, particularly in distinguishing them from conventional seafood. Disputes over labeling, similar to those seen in the lab-grown meat industry that led to a petition of the US Cattlemen’s Association to the US Department of Agriculture, Food Safety and Inspection Service, are likely to arise, potentially delaying market entry^91^. Labeling regulations are vital not only for ensuring consumer transparency but also for addressing broader social, economic, political, and cultural issues. These may vary significantly by region; some require only a basic declaration of the product type, while others enforce stricter guidelines to clearly distinguish cell-based products from their wild-caught or farmed counterparts. Trademarks add another layer of complexity, as they ensure the origin and quality of products.

The regulatory and legislative landscape for cell-based (sea)food remains fragmented and underdeveloped, with significant implications for the industry’s growth and global market access. Public funding could support the development of regulatory guidelines, helping to streamline approval processes and ensure consistency across the industry^42^.

## Conclusion

Cellular agriculture industry is presented by many as a revolutionary technology that can support traditional food industries in the quest to reduce hunger in the future. However, it is important to recognize that the first step to address this challenge is reducing food waste. In 2022, roughly 33% of food was wasted globally across the supply chain, including 13% from post-harvest stages up to the retail level and 19% across retail, food service, and households level^92^. Cellular agriculture seems to fall short in tackling issues such as poverty, social exclusion, and other factors contributing to unequal access to food^87^.

The cultured seafood industry, in particular, has the potential to complement fisheries and aquaculture in the pursuit of global food security. This review has highlighted the industry’s characteristics and limitations, emphasizing that it remains in its early stages of development. Specifically, it has examined the technological challenges, public health considerations, environmental implications, economic factors, regulatory landscape, and consumer acceptance of cultured seafood products.

As the industry matures, efforts to improve collaboration, enhance transparency, and harmonize regulations will be essential. Furthermore, careful consideration must be given to the potential economic displacement in traditional seafood markets and the environmental impact of large-scale production. Ultimately, the success of cultured seafood will depend on consumer acceptance. If these products can meet consumer expectations in terms of taste, quality, and affordability, they may establish a viable market alongside conventional fisheries and aquaculture.

More research is needed to explore the complex interplay of the six domains discussed here. As this field continues to develop, it will require a concerted effort from scientists, policymakers, and industry stakeholders to address these challenges and realize the full potential of cultured seafood.

## Data Availability

No datasets were generated or analyzed during the current study.
